# Comprehensive plasma cytokine and chemokine profiling in prurigo nodularis reveals endotypes in Type 2 inflammation

**DOI:** 10.1038/s41598-024-58013-x

**Published:** 2024-04-06

**Authors:** Hannah L. Cornman, Jaya Manjunath, Sriya V. Reddy, Jackson Adams, Ahmad Rajeh, Christeen Samuel, Aaron Bao, Ryan Zhao, Emily Z. Ma, Jason Shumsky, Thomas W. Pritchard, Brenda Umenita Imo, Alexander L. Kollhoff, Kevin K. Lee, Weiying Lu, Selina Yossef, Madan M. Kwatra, Shawn G. Kwatra

**Affiliations:** 1 Department of Dermatology, University of Maryland School of Medicine, Baltimore, MD USA; 2 Maryland Itch Center, University of Maryland School of Medicine, Baltimore, MD USA; 3 Department of Dermatology, Johns Hopkins University School of Medicine, Baltimore, MD 21231 USA; 4 Department of Anesthesiology, Duke University School of Medicine, Durham, USA

**Keywords:** Diseases, Skin diseases, Biomarkers

## Abstract

Prurigo nodularis (PN) is a chronic inflammatory skin disease that is associated with variability in peripheral blood eosinophil levels and response to T-helper 2 targeted therapies (Th2). Our objective was to determine whether circulating immune profiles with respect to type 2 inflammation differ by race and peripheral blood eosinophil count. Plasma from 56 PN patients and 13 matched healthy controls was assayed for 54 inflammatory biomarkers. We compared biomarker levels between PN and HCs, among PN patients based on absolute eosinophil count, and across racial groups in PN. Eleven biomarkers were elevated in PN versus HCs including interleukin (IL)-12/IL-23p40, tumor necrosis factor-alpha (TNF-α), Thymic stromal lymphopoietin (TSLP), and macrophage-derived chemokine (MDC/CCL22). Additionally, PN patients with AEC > 0.3 K cells/μL had higher Th2 markers (eotaxin, eotaxin-3, TSLP, MCP-4/CCL13), and African American PN patients had lower eosinophils, eotaxin, and eotaxin-3 versus Caucasian and Asian PN patients (p < 0.05 for all). Dupilumab responders had higher AEC (p < 0.01), were more likely to be Caucasian (p = 0.02) or Asian (p = 0.05) compared to African Americans, and more often had a history of atopy (p = 0.08). This study suggests that blood AEC > 0.3 K and Asian and Caucasian races are associated with Th2 skewed circulating immune profiles and response to Th2 targeted therapies.

## Introduction

Prurigo nodularis (PN) is a chronic, intensely pruritic inflammatory skin disease associated with a dramatic reduction in quality of life, increased health care utilization, and mortality^1–3^. Although primarily a cutaneous disease, PN involves substantial systemic inflammation, with elevated levels of γδT cells, iNK T cells, and various cytokines in the bloodstream^4–8^, as well as an association with many systemic comorbidities such as chronic kidney disease, cardiovascular disease, diabetes, and malignancy^9–11^. Insights into these systemic inflammation patterns can enhance our understanding of disease pathogenesis in PN.

The pathophysiology of PN is heterogenous, involving both neural and immune dysregulation^12^. Within immune dysregulation, individual variation can be substantial, with research highlighting differing involvement of the T-helper (Th)1, Th2, Th17, and Th22 immune axes in individual patients^13^. Heterogeneity is also evident in the clinical presentation, systemic comorbidity burden, and treatment response of PN patients^14^. In particular, African American patients demonstrate a genetic predisposition towards development of PN, bear a heavier comorbidity burden, and often exhibit more fibrotic and hyperkeratotic nodules compared to other racial groups^11,15–17^. Additionally, there is evidence of heterogeneity in response to immunomodulatory treatments, with some patient subgroups responding better to neural or immunomodulatory targeted therapies^18^. Our experience at the bedside has been that there is an extremely heterogeneous response in itch and nodule resolution among PN patients treated with dupilumab, currently the only FDA approved therapy for PN. Even in recent successful phase 3 clinical trials for PN treatment with dupilumab, a Th2 modulating therapy, over 40% of patients failed to achieve a clinically significant improvement in pruritus (quantified as a 4-point reduction in Worst Itch Numeric Rating Scale [WI-NRS] score)^19–21^. This suggests a variable response to these therapies and differing degrees of type 2 inflammation in PN subgroups.

Additional understanding of the heterogeneity in PN would improve disease management via precision medicine approaches. Based on the observed racial heterogeneity and variable response to Th2 targeting therapeutics in PN, we hypothesized that circulating immune profiles would differ based on race and clinically identifiable markers of type 2 inflammation, such as blood eosinophil levels. We thus analyzed circulating plasma cytokine levels in PN patients relative to healthy controls, in PN patients with versus without elevated eosinophils, and across racial groups as well as correlated these biomarkers with therapeutic response.

## Results

### Patient characteristics: plasma cytokine analysis cohort

The patient population for plasma cytokine analysis consisted of 56 patients with PN and 13 HCs without active pruritus. PN patients and HCs had similar distributions of age (55.8 ± 13 versus 50.1 ± 11.1 years), sex (66% versus 69% female), and race (39% versus 46% Caucasian, 54% versus 46% African American, and 7% versus 8% Asian). They also had similar proportions of patients with a history of atopy (45% versus 23%). Patients with PN had mean ± SD WI-NRS scores of 9.0 ± 1.2 and mean ± SD IGA scores of 3.2 ± 0.7. Demographics and characteristics for patient populations are detailed in Table 1.

**Table 1:** Baseline demographics of PN and HC patients in plasma cytokine analysis.

Characteristics	PN (n = 56), n (%)	Controls (n = 13), n (%)
Age (mean ± SD)	55.8 ± 13	50.1 ± 11.1
Sex		
Female	37 (66.1)	9 (69.2)
Male	19 (33.9)	4 (30.8)
Race		
White or caucasian	22 (39.3)	6 (46.2)
Black or african american	30 (53.6)	6 (46.2)
Asian	4 (7.1)	1 (7.6)
History of atopy	25 (44.6)	3 (23.1)
Worst itch numeric rating scale score (mean ± SD)	9.0 ± 1.2	0 ± 0
Investigator's global assessment score (mean ± SD)	3.2 ± 0.7	0 ± 0

### Analysis of plasma biomarkers in PN versus controls showed elevated inflammatory markers in PN

**Figure 1:**
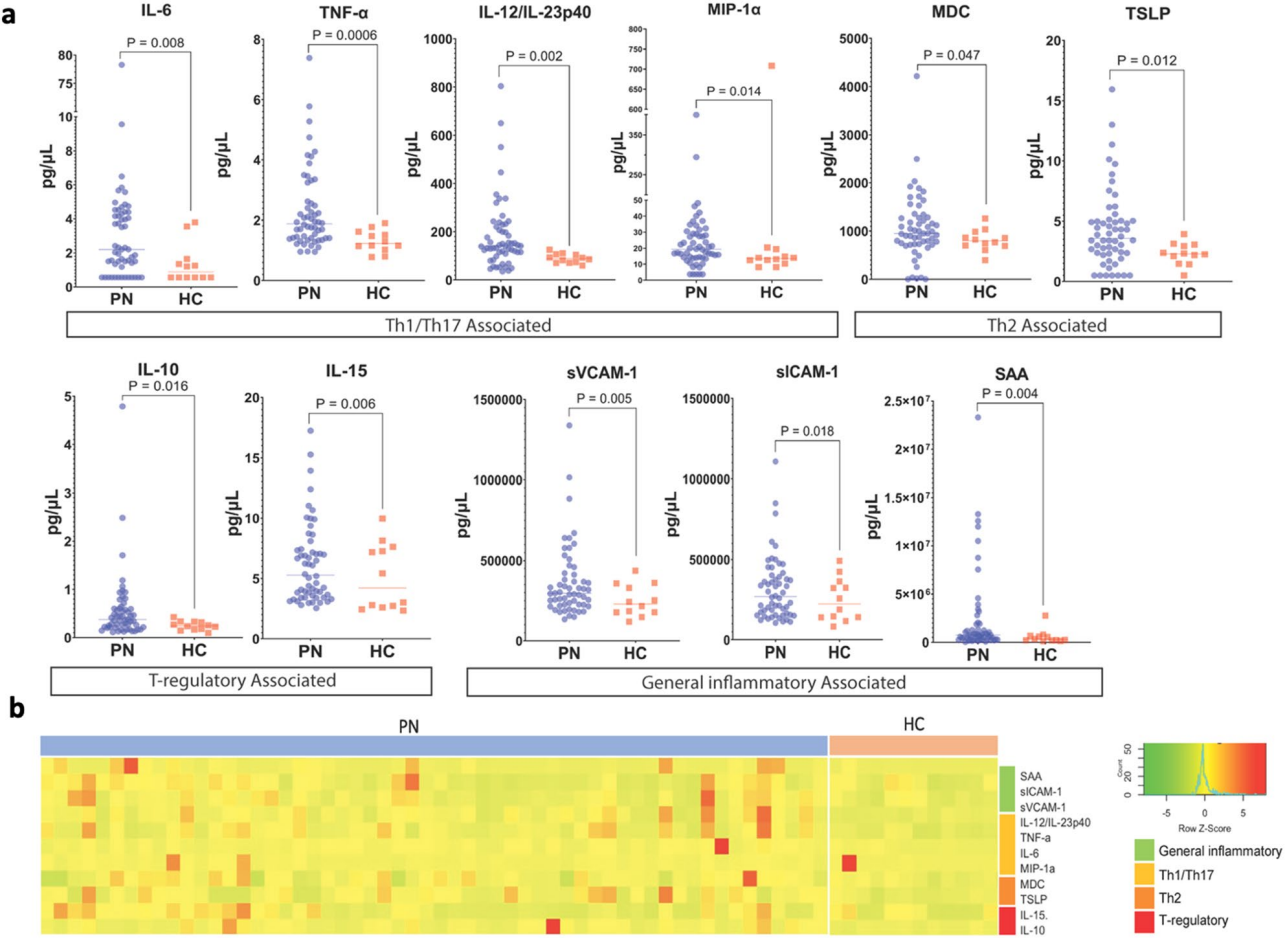
Plasma biomarker analysis of PN versus HC samples. (**a**) Dot plots showing plasma levels of biomarkers significantly elevated in PN versus HC samples, labeled by their association with immune axes, T-regulatory cells, or general inflammation. Unadjusted P-value is shown. (**b**) Heatmap of z-score transformed biomarker levels for each patient, delineated by PN and HC. *PN* Prurigo nodularis, *HC* healthy controls, *Th* T-helper.

Comparison of 54 biomarkers in PN versus HC plasma revealed significant elevation of 11 markers including interleukin (IL)-6 (P = 0.008, q = 0.057), tumor necrosis factor alpha (TNF-α) (P = 0.0006, q = 0.023), IL-12/IL-23p40 (P = 0.002, q = 0.042), Macrophage inflammatory protein-1 alpha (MIP-1α/CCL3) (P = 0.014, q = 0.069), Macrophage-derived chemokine (MDC/CCL22) (P = 0.047, q = 0.174), Thymic stromal lymphopoietin (TSLP) (P = 0.012, q = 0.069), IL-10 (P = 0.016, q = 0.073), IL-15 (P = 0.006, q = 0.05), Soluble Vascular Cell Adhesion Molecule-1 (sVCAM-1) (P = 0.005, q = 0.05), Soluble intercellular adhesion molecule-1 (sICAM-1) (P = 0.018, q = 0.075), and serum amyloid A (SAA) (P = 0.004, q = 0.05) (Fig. 1). No biomarkers were significantly decreased in PN compared to HC plasma.

### PN patients with elevated AEC have increased levels of Th2 associated inflammatory markers

**Figure 2:**
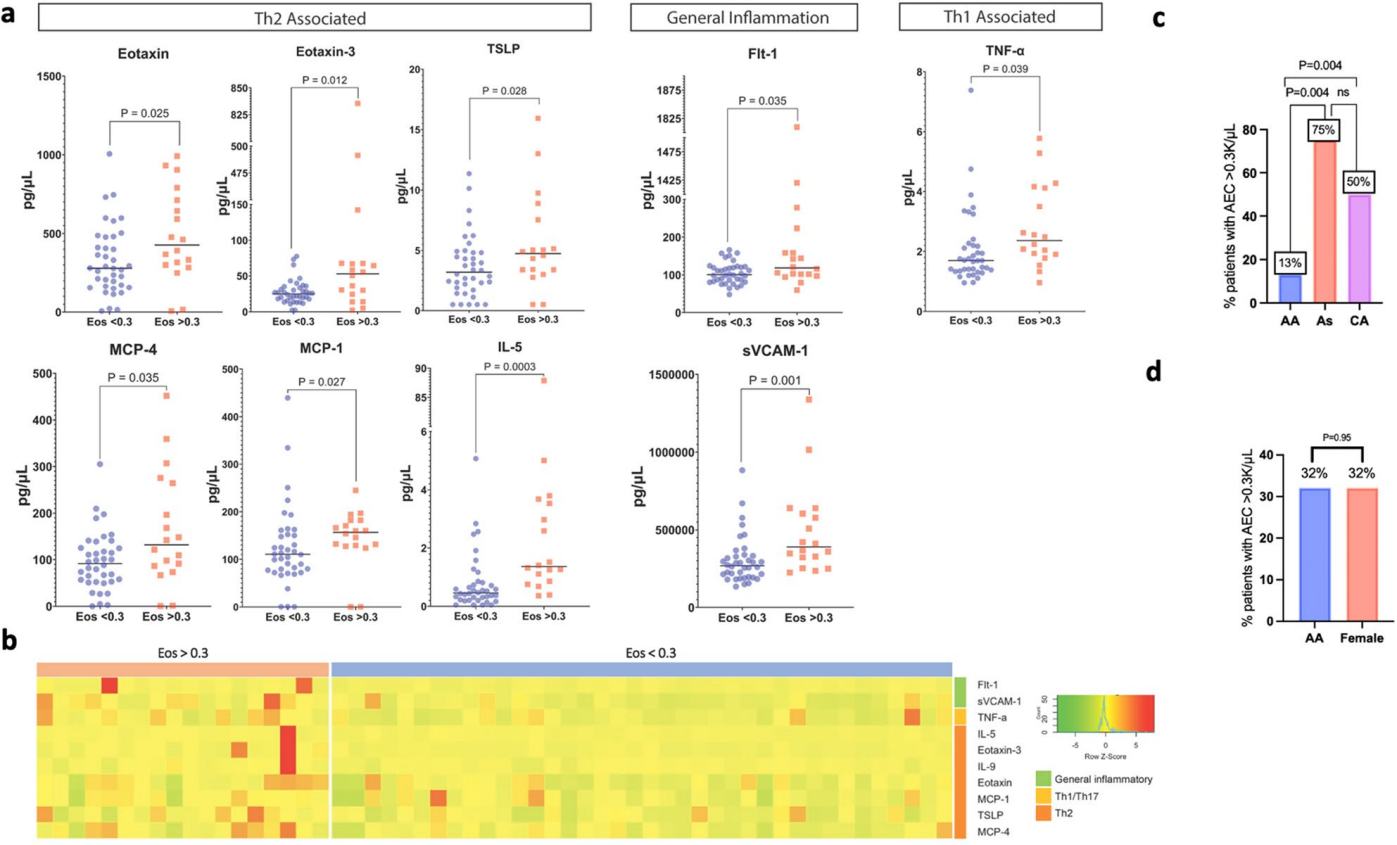
Plasma biomarker analysis of PN patients with elevated eosinophils (AEC > 0.3 K cells/μL) versus non-elevated eosinophils (AEC < 0.3 K cells/μL). (**a**) Dot plots showing plasma levels of biomarkers significantly elevated in cohort with elevated eosinophils, labeled by their association with immune axes or general inflammation. (**b**) Heatmap of z-score transformed biomarker levels for each patient, delineated by PN patients with AEC greater than or less than 0.3 K cells/μL. (**c**) Bar graph showing the percentage of patients in each racial category with AEC > 0.3 K cells/μL. (**d**) Bar graph showing the percentage of patients in males and females with AEC > 0.3 K cells/μL. *AA* African American, *As* Asian, *CA* Caucasian.

A total of 18 PN patients in the plasma cytokine cohort (32%) had elevated AEC (> 0.3 K cells/μL). Compared to those without elevated AEC, (n = 38, 68%), this subgroup had significantly higher levels of 9 biomarkers, predominantly consisting of cytokines and chemokines involved in type 2 inflammation such as eotaxin (P = 0.025, q = 0.16), eotaxin-3 (P = 0.012, q = 0.15), TSLP (P = 0.028, q = 0.16), monocyte chemotactic protein-4 (MCP-4/CCL13) (P = 0.035, q = 0.16), Monocyte chemoattractant protein-1 (MCP-1/CCL2) (P = 0.027, q = 0.16), and IL-5 (P = 0.0003, q = 0.013). They also had higher levels of TNF-α (P = 0.039, q = 0.16), Flt-1/vascular endothelial growth factor receptor 1 (VEGFR-1) (P = 0.035, q = 0.16), and sVCAM-1 (P = 0.001, q = 0.028) (Fig. 2a,b). A significantly larger proportion of Asian and Caucasian patients had elevated AEC compared to African American patients (75% versus 13%, P = 0.004 and 50% versus 13%, P = 0.004, respectively), and there was no significant difference in the proportion of males and females who had elevated AEC (32% versus 32%, P = 0.95) (Fig. 2c,d).

### African American PN patients have lower eosinophil counts and Th2 related biomarkers than Asian and Caucasian PN patients

**Figure 3:**
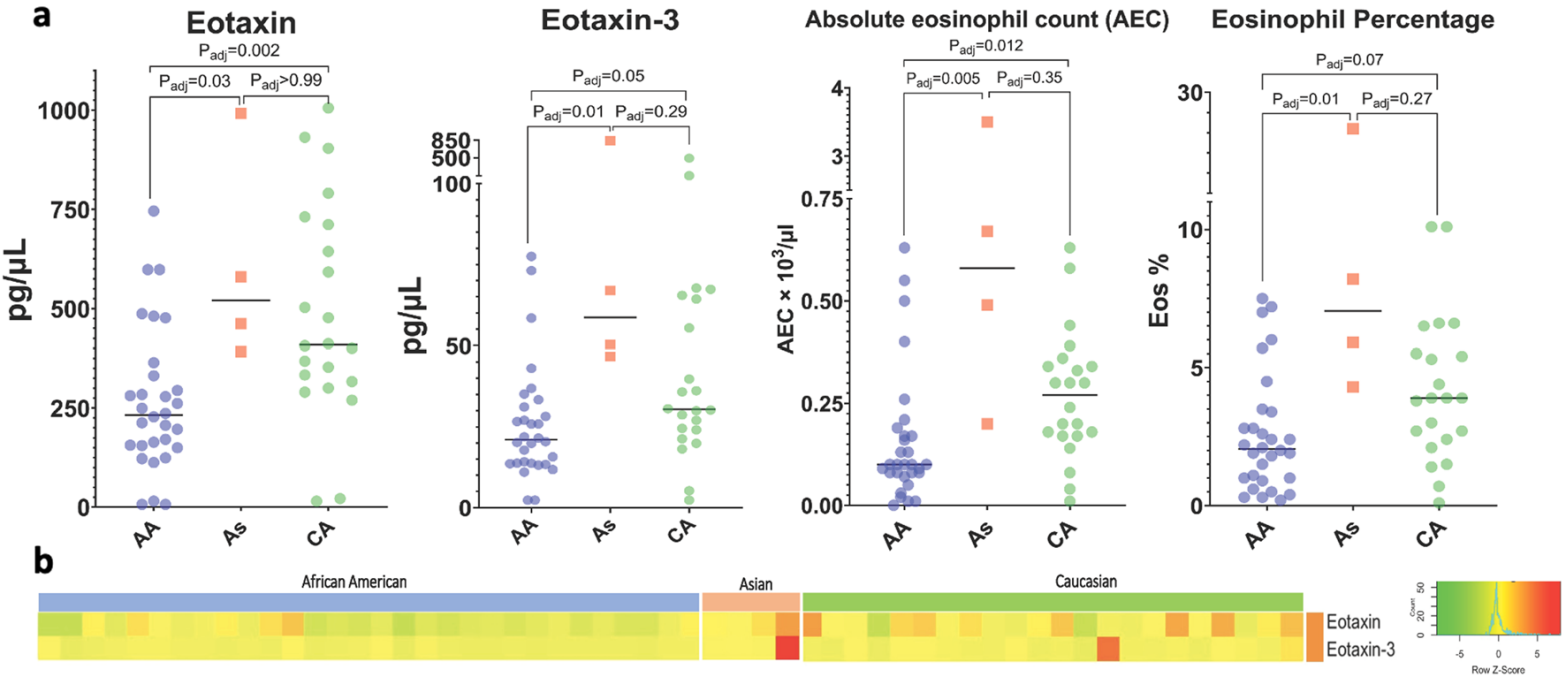
Plasma biomarker analysis of African American versus Asian versus Caucasian PN patients. (**a**) Dot plots showing plasma levels of biomarkers with significant differences between racial cohorts. (**b**) Heatmap of z-score transformed biomarker levels for each patient, delineated by race. *AA* African American, *As* Asian, *CA* Caucasian. Adjusted P-values (from Kruskall–Wallis) are shown.

Comparison of biomarker levels across races revealed lower levels of eotaxin and eotaxin-3 in African American (AA) compared to Caucasian (CA) (P_adj_ = 0.002 and P_adj_ = 0.05, respectively) and Asian (As) (P_adj_ = 0.03 and P_adj_ = 0.01, respectively) PN patients (Fig. 3). There was no significant difference in either of these cytokines between Caucasian and Asian patients (P_adj_ > 0.99 and P_adj_ = 0.29, for eotaxin and eotaxin-3, respectively). African Americans also had lower levels of AEC and eosinophil percentage compared to Caucasian (P_adj_ = 0.012 and P_adj_ = 0.07, respectively) and Asian (P_adj_ = 0.005 and P_adj_ = 0.01, respectively) PN patients. There was no significant difference in AEC or eosinophil percentage between Caucasian and Asian patients (P_adj_ > 0.35 and P_adj_ = 0.27, respectively).

### Dupilumab response in PN is associated with higher AEC, Asian or Caucasian race, and atopy

**Figure 4:**
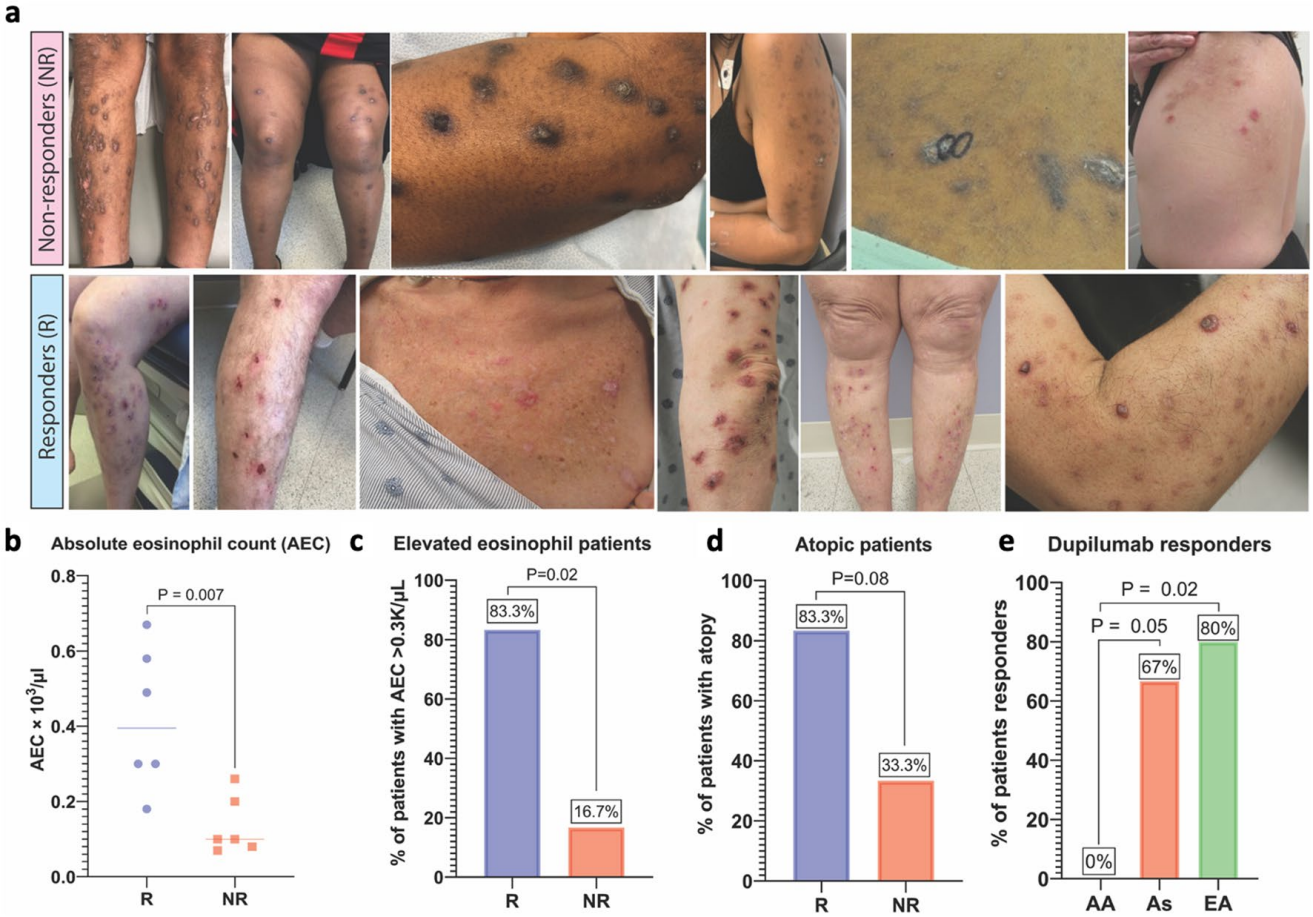
Clinical characteristics and biomarkers of PN patients with and without improvement on dupilumab therapy. (**a**) Representative clinical photos of PN patients who achieved clinically significant improvement in itch (responders) and those who did not (non-responders) after at least 6 months of dupilumab therapy. (**b**) AEC of dupilumab responders (R) versus non-responders (NR). (**c**) Percentage of patients with AEC > 0.3 K cells/μL in dupilumab R versus NR. (**d**) Percentage of patients with a history of atopy in dupilumab R versus NR. (**e**) Percentage of patients in each racial category that were dupilumab responders. *AA* African American, *As* Asian, *CA* Caucasian.

Given that elevated AEC and Asian or Caucasian race were associated with higher levels of type 2 inflammation markers, we predicted that patients with these clinical features might demonstrate better response to dupilumab, an IL-4 receptor alpha (IL-4Rα) antagonist recently approved for the treatment of PN^22^. Retrospective chart review identified 12 patients treated with dupilumab who met all inclusion and exclusion criteria. The mean ± SD age of all patients was 55.8 ± 10.4. Four patients (33%) were African American, 5 (42%) were Caucasian, and 3 (25%) were Asian. Six patients (50%) experienced clinically significant improvement (labeled as responders), defined as a sustained ≥ 4-point decrease in WI-NRS score, and 6 patients did not (labeled as non-responders). Representative clinical photos of responders (R) and nonresponders (NR) are shown in Fig. 4a. Four patients (33.3%) achieved ‘clear’ or ‘almost clear’ skin (IGA score of 0 or 1) in addition to a 4-point drop in WI-NRS. There was no significant difference between responders and non-responders in baseline WI-NRS (9.7 ± 0.5 versus 9.0 ± 1.3, p = 0.47) or IGA (3.0 ± 0.6 versus 3.7 ± 0.5, P = 0.18). Compared to non-responders, dupilumab responders were older (61.8 ± 5.6 versus 49.7 ± 10.8, P = 0.03), had a higher AEC (0.42 ± 0.2 K/μL versus 0.13 ± 0.08 K/μL, P < 0.01), were more likely to have an AEC > 0.3 K cells/μL (83.3% versus 16.7%, P = 0.02), and were more likely to have a history of atopy (83.3% versus 33.3%, P = 0.08) (Fig. 4b–d). A smaller proportion of African Americans responded to dupilumab as compared to Asians (0% versus 66.7%, P = 0.05) and Caucasians (0% versus 80%, p = 0.02) (Fig. 4e). Adverse events included new-onset psoriasis in two responders, neither of which required drug discontinuation. The demographics and clinical characteristics of these patients can be found in Table 2.

**Table 2:** Demographics and clinical characteristics of patients in retrospective cohort study.

Characteristics	All patients (n = 12), n (%)	Responders (n = 6), n (%)	Non-responders (n = 6), n (%)	P-value
Age (mean ± SD)	55.8 ± 10.4	61.8 ± 5.6	49.7 ± 10.8	**0.03**
Sex				0.221
Female	8 (66.7)	3 (50.0)	5 (69.2)	
Male	4 (33.3)	3 (50.0)	1 (30.8)	
Race				**0.046**
White or Caucasian	5 (41.7)	4 (66.7)	1 (20.0)	
Black or African American	4 (33.3)	0 (0.0)	4 (100.0)	
Asian	3 (25.0)	2 (33.3)	1 (33.3)	
Worst itch numeric rating scale Score (mean ± SD)	9.3 ± 1.0	9.7 ± 0.5	9.0 ± 1.3	0.47
Investigator's global assessment score (mean ± SD)	3.3 ± 0.7	3.0 ± 0.6	3.7 ± 0.5	0.18
Eosinophil percentage (%)	4.3 ± 2.9	5.5 ± 3.1	3.0 ± 2.1	0.18
Absolute eosinophil count (AEC) (K cells/μL)	0.28 ± 0.2	0.42 ± 0.2	0.13 ± 0.08	** < 0.01**
AEC > 0.3 K cells/μL	6 (50.0)	5 (83.3)	1 (16.7)	**0.02**
History of atopy	7 (58.3)	5 (83.3)	2 (33.3)	0.08

Significant values are in bold.

## Discussion

PN has been described as a clinically heterogenous disease, but there is a need for a deeper understanding of the underlying molecular mechanisms driving this heterogeneity. These distinct pathophysiological mechanisms define endotypes, or subtypes of PN, with different clinical features. In this study, we build on preliminary studies by employing a large PN patient cohort to identify numerous cytokines and chemokines elevated in PN as compared to healthy controls which have not been previously reported. Furthermore, this analysis identified distinct endotypes of PN, defined by identifiable clinical characteristics such as race and blood eosinophil levels, and a correlation between these clinically identifiable features and response to dupilumab treatment.

In comparing cytokine levels of patients with PN to healthy controls, we found that IL-6, TNF-α, IL-12/IL23p40, MIP-1α/CCL3, MDC/CCL22, TSLP, IL-10, IL-15, sVCAM-1, sICAM-1, and SAA were significantly elevated. Prior studies have found elevated levels of CUB domain-containing protein 1 (CDCP1), Monocyte Chemotactic Protein 3 (MCP-3), IL-13, periostin, IL-31, IL-6, caspase 8, β-endorphin, and autotaxin in the blood of PN patients^5–7,23–25^. Several Th1/Th17 associated cytokines that were elevated in PN compared to HCs and have not been previously linked to PN include MIP-1α/CCL3 and IL-15. MIP-1α/CCL3 is a chemotactic chemokine secreted predominantly by macrophages. It is involved in recruiting inflammatory cells (macrophages, polymorphonuclear leukocytes, eosinophils), wound healing, and activation of osteoclasts and bone resorption^26^. IL-15 is involved in the development and activation of natural killer (NK) and NK T-cells, as well as γ/δT cells, all cell types which have been implicated in the pathogenesis of PN by prior studies^4,27^.

Additional dysregulated markers in PN versus HCs included Th2 associated cytokines MDC/CCL22, TSLP, and IL-10. MDC/CCL22 acts as a Th2 skewed chemoattractant, upregulates TARC/CCL17 production, and has been found to correlate with atopic dermatitis disease activity^28–30^. TSLP promotes secretion of periostin in keratinocytes via the JAK-STAT pathway and to induce M2 macrophage differentiation and IL-31 production in coordination with periostin and basophils^31,32^. IL-10 is an anti-inflammatory cytokine that is produced by Th2 cells and monocytes, known to be elevated in AD, and functions to decrease Th1 associated cytokine expression^33,34^. Finally, there were also increased levels of general inflammatory molecules in PN plasma, such as sVCAM-1 and sICAM-1 which are known to facilitate leukocyte adhesion and migration across the blood vessel endothelium^35,36^, as well as the acute phase reactant SAA, which promotes inflammation by activating the inflammasome cascade, inducing synthesis of pro-inflammatory cytokines, and acting as a chemotactic for neutrophils and mast cells^37^. Each of these general inflammatory markers are known biomarkers of cardiovascular disease, which disproportionately affects patients with PN^3,38,39^. This lends support to the hypothesis that systemic inflammation in PN contributes to development of cardiovascular disease in this population.

We also discovered higher levels of predominantly type 2 inflammation-related cytokines in PN patients with elevated AEC versus those without, suggesting a Th2 dominant PN endotype that can be distinguished clinically by peripheral blood eosinophil level. The cytokines increased in PN patients with high AEC included eotaxin, eotaxin-3, MCP-1, and MCP-4, which are all chemokines produced by endothelial cells that regulate eosinophil chemotaxis, though MCP-1 and MCP-4 also have actions on monocytes and T cells^40–42^. IL-5 similarly plays a pivotal role in promoting the differentiation, recruitment, survival, and activation of eosinophils^43^. TSLP, in addition to its aforementioned roles, activates eosinophils to produce Th2 associated cytokines, a function which is enhanced by TNF-α^44^. Thus, in this scenario, TNF-α is associated with a Th2 rather than a Th1 immune response. A prior study using unsupervised clustering algorithms found that a discrete subset of PN patients had elevated Th2 markers (IL-13 and IL-5), supporting our claims that such an endotype exists^6^. Our results expand on this study by detecting more Th2 associated cytokines elevated in this subset of PN patients, and finding a clinically available biomarker, eosinophil levels, that can aid in identifying this subset of patients. Eosinophils are innate immune leukocytes which have long been associated with the Th2 immune response^45^, and therefore eosinophil levels in peripheral blood or sputum have been used as clinical biomarkers of Th2 dominant endotypes in numerous diseases such as asthma, chronic obstructive pulmonary disease (COPD), atopic dermatitis, and chronic pruritus of unknown origin (CPUO)^46–49^. The presence of a Th2 endotype is clinically useful in predicting therapeutic response to immunologic therapeutic strategies. For example, in CPUO increased circulating blood eosinophils (> 4% or > 0.30 K/μL) was found to predict favorable response to immunomodulatory therapy^48^. Additionally, in asthma, treatment eligibility criteria for IL-5 targeting monoclonal antibodies or dupilumab includes the presence of elevated Th2 biomarkers that are predictive of a more likely patient response, such as blood eosinophils ≥ 0.30 K/μL or FeNO ≥ 25 ppb^50^.

Our studies also revealed that Caucasian and Asian PN patients were more likely to have elevated AEC and had higher levels of Th2 associated inflammation markers (eotaxin, eotaxin-3, AEC, and eosinophil percentage) than their African American counterparts, supporting the existence of a distinct African American PN endotype with lesser systemic Th2 immune axis involvement. Previous studies have also found evidence of racially based molecular heterogeneity in PN. Fukushi et al. showed Th2 as a principal driver of PN in a Japanese cohort^51^, whereas Belzberg et al. found that PN lesional skin was characterized by Th22/Th17 axis overexpression in a predominantly African American population^4^. Belzberg et al. also observed heightened Th2 axis polarization in European PN patients^4^. Finally, Sutaria et al. found a distinct cluster of mostly African American PN patients who expressed increased levels of circulating pro-inflammatory cytokines involved in the Th1, Th2, Th17, and Th22 immune axes (IL-1α, IL-4, IL-5, IL-6, IL-10, IL-17A, IL-22, IL-25, and IFN-α) as compared to a cluster with mostly Caucasian PN patients^18^. These types of race-based endotypes are well established in other dermatologic conditions such as AD, in which Asian patients have accentuated polarity of the Th22/Th17 pathways, and African American patients have distinct attenuation of Th17/Th1 immune axes^52^. Additionally, our results are consistent with prior literature which has shown Black ethnicity to be associated with lower average eosinophil counts and higher average lymphocyte counts in patients with cardiovascular disease^53^.

Lastly, to shed light on the clinical implications of our plasma cytokine studies, we explored whether Caucasian or Asian ethnicity, or elevated blood eosinophil levels, all identified as clinical traits correlated with increased Th2 immune axis markers, were associated with enhanced response to dupilumab treatment. Overall, in a retrospective chart review of 12 patients treated with at least 6 months of dupilumab, we observed a 50% response rate to dupilumab, highlighting the variability in response to dupilumab among PN patients.

Consistent with our plasma cytokine findings, we found that higher AEC, Caucasian or Asian race, and a clinical history of atopy were associated with achieving a clinically meaningful reduction in pruritus on dupilumab therapy. Given that dupilumab targets type 2 inflammation by blocking IL-4Rα and inhibiting IL-4 and IL-13 signaling, this is consistent with our findings of increased Th2 markers in our plasma cytokine analysis (high AEC and Caucasian/Asian race) are predictive of improved responsiveness to this drug^22^. Patients who are not responsive to dupilumab thus might benefit from treatments that target broader immune axes or mechanisms of fibrosis and neural dysregulation.

Limitations of this study include small sample size, especially in the retrospective review of dupilumab response and in the number of Asian patients included. Larger registry studies with an emphasis on including larger numbers of patients with different race and ethnicity are required to expand these findings to wider, diverse patient populations and to improve the robustness of sub-analyses. Additionally, there is a need for studies focused on the efficacy of dupilumab in patients of African American or Black race. This is warranted given the findings of decreased response in this patient population in our retrospective analysis, the disproportionate prevalence and morbidity of PN among African Americans^15,54^, and phase 3 clinical trials for dupilumab included only 5% (8/160) African American patients, and were enriched for Caucasian and Asian PN patients^19^. Observation of only African American, Asian, and Caucasian patients limits broad generalizations, particularly about other races. The cross-sectional design precludes inferences about causality. Finally, this cytokine panel may not encompass the entire range of circulating biomarkers contributing to the pathogenesis of PN.

In summary, our findings suggest novel circulating biomarkers elevated in PN patients, as well as unique endotypes of PN which feature variable levels of Th2 immune dysregulation, are clinically identifiable by eosinophil levels or race, and describe the variable response to dupilumab therapy among PN patients. These findings advance our current understanding of heterogeneity in PN clinical presentation and treatment response and highlight the importance for stratifying for these blood biomarkers and including diverse patient populations in clinical trials.

## Methods and materials

**Figure 5:**
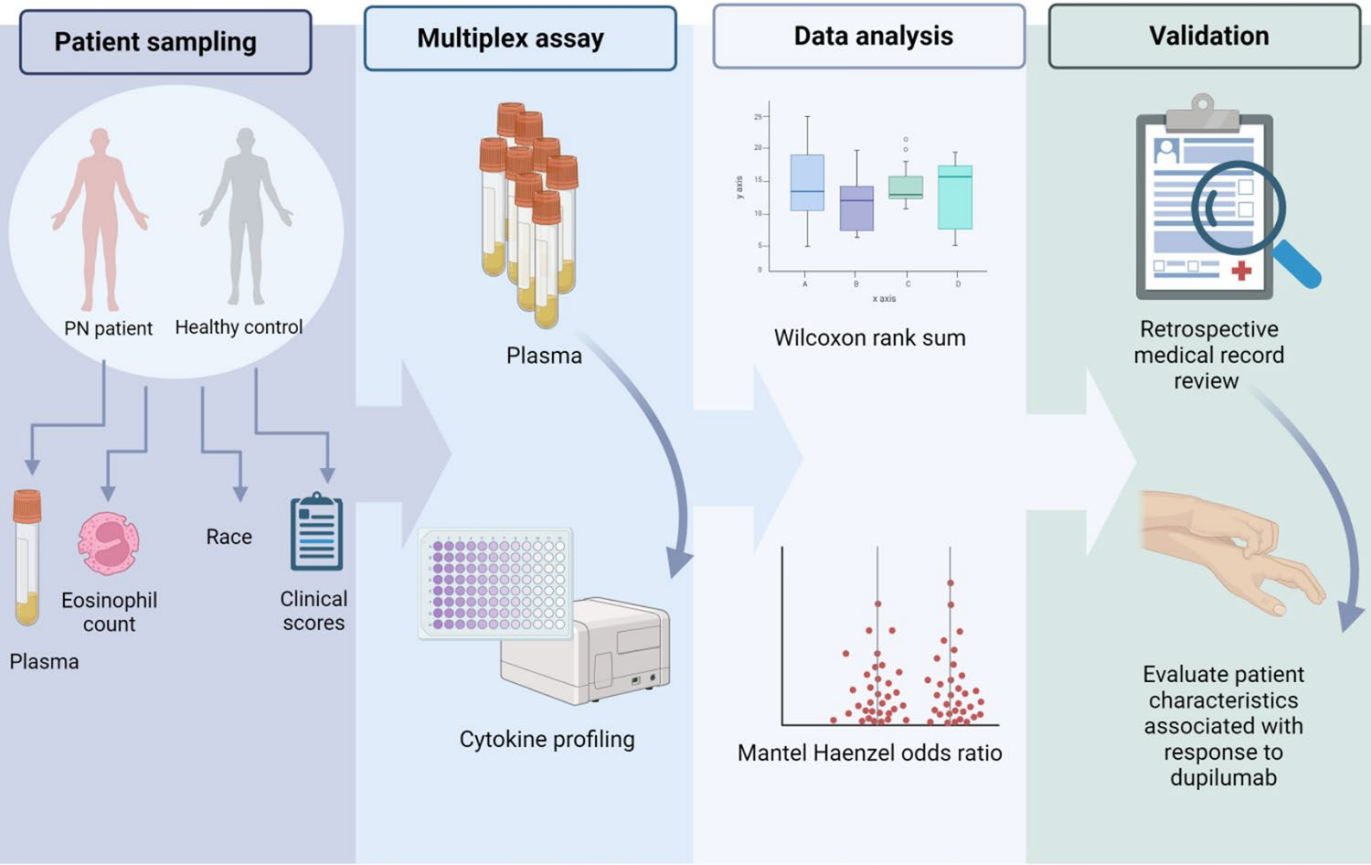
Schematic overview of study design.

An overview of the study design is depicted in Fig. 5.

### Study population

For this study we enrolled 56 patients with prurigo nodularis (PN) and 13 age, sex, and race matched healthy controls (HCs). Inclusion criteria for PN patients were modeled after previous clinical trials of PN, including diagnosis of PN by a board-certified dermatologist, at least 20 nodules on bilateral regions of the body, and a WI-NRS score of at least 7^55^. Demographic and clinical characteristics of patients were collected at the time of blood draw. A complete blood count with differential conducted within 3 months of the blood draw for research was used to obtain the absolute eosinophil count (AEC) and eosinophil percentage values. Disease severity was assessed and documented using validated tools such as the WI-NRS and the Investigator’s Global Assessment (IGA)^56^. A history of atopy was defined as having a documented clinical history of two of three of the following atopic diatheses: asthma, allergic rhinitis, or atopic dermatitis/eczema. An elevation in eosinophils was defined as > 0.3 K cells/μL in absolute eosinophil count (AEC), as this cutoff has been used to predict therapeutic response and exacerbation risk in other Th2 associated diseases such as asthma and chronic obstructive pulmonary disease (COPD)^57^.

### Blood processing and cytokine quantification

Approximately 2 mL of whole blood was collected from each study participant and partitioned via centrifugation (for 10 min at 1960 g) for the isolation of plasma, peripheral blood mononuclear cells, and serum. Plasma was then carefully separated, avoiding the buffy coat, and transferred into separate tubes to create 1 mL aliquots. Next, the aliquots were frozen slowly at a temperature of − 80 °C for 24 to 72 h and transferred into the liquid nitrogen vapor phase for storage.

Plasma samples were analyzed for the presence of 54 proinflammatory markers including cytokines, chemokines, Th17 mediators, angiogenetic and vascular injury factors using the V-PLEX Human Biomarker 54-Plex Kit (MesoScale Diagnostics, Rockville, MD) Human Biomarker 54-Plex Kit consisting of Proinflammatory Panel 1, Cytokine Panel 1, Cytokine Panel 2, Chemokine Panel 1, Th17 Panel 1, Angiogenesis Panel 1 and the Vascular Injury Panel 2 according to the manufacturer’s instructions. All samples were run in two batches of 54-plex kits. 8 random technical duplicates were used to extrapolate the average CV. The presence of the analytes was measured in 96 well plates using the Quickplex SQ 120 instrument (Mesoscale) via electrochemiluminescence. Sample concentrations were calculated following extrapolation of the analyte standard curves with a four-parameter logistic fit using MSD Workbench 3.0 software.

### Longitudinal analysis of clinical response among PN patients to dupilumab therapy

We conducted a retrospective medical record review of PN patients seen at the Johns Hopkins Itch Center to evaluate real world evidence of PN patient characteristics associated with response to dupilumab. Inclusion criteria included fulfillment of the formal criteria for PN diagnosis as determined by an expert consensus panel^58^, treatment with dupilumab for at least 6 months^19^, and availability of WI-NRS scores to determine if there was a clinically significant treatment response. Exclusion criteria included treatment with additional systemic medications during the time of dupilumab therapy, and uncontrolled systemic conditions, such as diabetes, thyroid disorders, cardiac disease, and rheumatologic disease. All patients received a 600 mg loading dose followed by 300 mg biweekly dosing. Patients were followed for up to 36 months after starting treatment. Data was collected on the WI-NRS scores as a measure of response to therapy. Baseline demographic characteristics and laboratory values such as AEC and eosinophil percentage were collected via complete blood count with differential, as described above. Patients were assigned to responder versus non-responder groups based on whether they had clinically significant improvement, defined as a ≥ 4-point decrease in WI-NRS score after 6 months of therapy, without clinical rebound at the time of medical record review.

### Statistical analysis

Cleaning and imputation were performed on the raw plasma cytokine data utilizing methods adopted from a previous study^18^. Briefly, biomarkers with > 33% of samples below the detection limit (DL) were dichotomized into binomial variables with values below the DL as negative and values above the DL as positive (Supplementary Table 1). For all other biomarkers, values below the DL were imputed with half the DL. The samples were run in two batches, and to reduce the potential effect of batches on analysis results, the following statistical analysis methods were used. For continuous variables, cytokine concentrations were compared by performing nonparametric rank sum test analyses separately in the two batches, and then combining the two p-values using the weighted sum of z. For dichotomous variables, cytokine concentrations were compared by performing Mantel Haenzel odds ratio analyses. For comparison of blood eosinophil levels (absolute eosinophil count, eosinophil percentage) and demographic characteristics, continuous variables were compared using the Mann–Whitney *U* test when there were 2 comparator groups and the Kruskall-Wallis test when there were 3 or more comparator groups. Categorical variables were compared using Pearson’s χ^2^ test. For cytokine analyses, false discovery rates (FDR) were calculated with the Benjamini–Hochberg procedure to control for multiple comparisons. Significance was determined by p-value < 0.05 and a false discovery rate (FDR) q < 0.2. Relaxed FDRs are often used in genetic and cytokine assay studies in which prior knowledge was used to select candidate genes and cytokines which are likely to have significant results^59–61^.

### Study approval

The approval for this study was obtained from the Johns Hopkins Institutional Review Board (IRB00231694). Written informed consent was obtained from each participant and is on file. All experiments were performed in accordance with regulations.

### Supplementary Information

Supplementary Table 1.

## Data Availability

The data analyzed in this study is available upon reasonable request from the corresponding author.
