# Supplementary material for: Comprehensive plasma cytokine and chemokine profiling in prurigo nodularis reveals endotypes in Type 2 inflammation
Source: Sci Rep. 2024 Apr 6;14:8098. doi: 10.1038/s41598-024-58013-x (PMC10998852; doi:10.1038/s41598-024-58013-x)
Supplement: Supplementary file 1 — Supplementary Table 1. [file 41598_2024_58013_MOESM1_ESM.docx]

**Supplementary Table 1. Lower Detection Limits and Data Classification for Cytokines**

| **Cytokine** | **Lower Detection Limit (pg/μL)** | **Data Classification** |
| --- | --- | --- |
| GM-CSF | 0.204153591 | Binomial |
| IL-12/IL-23p40 | 0.406296867 | Continuous |
| IL-15 | 0.176887268 | Continuous |
| IL-16 | 2.634162748 | Continuous |
| IL-17A | 0.414224964 | Continuous |
| IL-1a | 0.241058357 | Binomial |
| IL-5 | 0.089147896 | Continuous |
| IL-7 | 0.270892119 | Continuous |
| TNF-B | 0.128051766 | Binomial |
| VEGF-A | 0.753420756 | Continuous |
| IL-17A/F | 3.461670422 | Binomial |
| IL-17B | 1.722833804 | Continuous |
| IL-17C | 1.042075347 | Continuous |
| IL-17D | 4.073962175 | Continuous |
| IL-1RA | 1.568306328 | Continuous |
| IL-3 | 18.39380021 | Binomial |
| IL-9 | 0.321651425 | Continuous |
| TSLP | 1.002138932 | Continuous |
| IFN-g | 0.625271863 | Continuous |
| IL-10 | 0.031965399 | Continuous |
| IL-12p70 | 0.206225804 | Binomial |
| IL-13 | 2.024901353 | Binomial |
| IL-1B | 0.051053165 | Continuous |
| IL-2 | 0.497684371 | Binomial |
| IL-4 | 0.047570068 | Binomial |
| IL-6 | 1.119239452 | Continuous |
| IL-8 | 0.058589389 | Continuous |
| TNF-a | 0.12220162 | Continuous |
| IL-21 | 6.975774792 | Binomial |
| IL-22 | 0.388789111 | Binomial |
| IL-23 | 3.567394295 | Binomial |
| IL-27 | 53.8677591 | Continuous |
| IL-31 | 0.277176015 | Binomial |
| MIP-3a | 1.044375032 | Continuous |
| Eotaxin | 13.60984199 | Continuous |
| Eotaxin-3 | 4.648786416 | Continuous |
| IP-10 | 0.419568025 | Continuous |
| MCP-1 | 0.167197049 | Continuous |
| MCP-4 | 0.219888491 | Continuous |
| MDC | 6.9054776 | Continuous |
| MIP-1a | 7.338130497 | Continuous |
| MIP-1B | 2.101844967 | Continuous |
| TARC | 0.630310247 | Continuous |
| bFGF | 0.468269719 | Continuous |
| Flt-1 | 2.10401608 | Continuous |
| PIGF | 0.285334146 | Continuous |
| Tie-2 | 81.71160812 | Continuous |
| VEG-F A | 2.137370898 | Continuous |
| VEGF-C | 25.67567104 | Continuous |
| VEGF-D | 26.12700607 | Continuous |
| CRP | 5332.723556 | Continuous |
| SAA | 30538.68236 | Continuous |
| sICAM-1 | 1715.370768 | Continuous |
| sVCAM-1 | 15053.18251 | Continuous |
